# Subjective Cognitive Complaints in Mild Traumatic Brain Injury: Association with Cognitive Test Performance and Protective Psychological Factors

**DOI:** 10.1093/arclin/acaf055

**Published:** 2025-06-13

**Authors:** Kaisa Mäki, Taina Nybo, Marja Hietanen, Antti Huovinen, Ivan Marinkovic, Susanna Melkas

**Affiliations:** Department of Neuropsychology, University of Helsinki and Helsinki University Hospital, PO Box 302, FI-00029 Helsinki, Finland; Department of Neuropsychology, University of Helsinki and Helsinki University Hospital, PO Box 302, FI-00029 Helsinki, Finland; Department of Neuropsychology, University of Helsinki and Helsinki University Hospital, PO Box 302, FI-00029 Helsinki, Finland; Department of Neurology, University of Helsinki and Helsinki University Hospital, PO Box 340, FI-00029 Helsinki, Finland; Department of Neurology, University of Helsinki and Helsinki University Hospital, PO Box 340, FI-00029 Helsinki, Finland; Department of Neurology, University of Helsinki and Helsinki University Hospital, PO Box 340, FI-00029 Helsinki, Finland

**Keywords:** Head injury, Traumatic brain injury

## Abstract

**Objective:**

To examine subjective cognitive complaints (SCC) in patients with mild traumatic brain injury (mTBI), and to explore the associations between SCC, cognitive test performance and protective psychological factors.

**Method:**

A sample of patients with mTBI (n = 99) or orthopedic injury (OI; n = 34) prospectively recruited and assessed 3 months post-injury. All participants underwent a neuropsychological test battery and completed self-report measures on SCC, psychological resilience, perceived social support, depressive symptoms, fatigue, and pain.

**Results:**

27.3% of the patients with mTBI and 17.6% of the OI controls endorsed at least some SCC. The two groups did not differ significantly in their SCC endorsement. Within the mTBI group, patients with and without SCC did not differ significantly in their cognitive test performance in majority of the cognitive domains examined. Patients with SCC reported lower psychological resilience (*p* = .005) and perceived social support (*p* = .009) than the non-SCC group.

**Conclusions:**

This study provides support for the notion that SCC following mTBI are not consistently related to cognitive test performance deficits and further suggests SCC may associate with perceived social support and psychological resilience. These findings highlight the importance of considering psychological factors in the clinical assessment and intervention planning of patients presenting with SCC after mTBI.

## INTRODUCTION

With an estimated 100–300 hospital-treated cases per 100,000 persons per year worldwide, mild traumatic brain injury (mTBI) is a major public health concern (Cassidy et al., 2004; Nguyen et al., 2016). Although recovery is quick in many cases, a considerable proportion of patients report experiencing symptoms for months or even years after injury (Cancelliere et al., 2023; Machamer et al., 2022; Theadom et al., 2018).

Subjective cognitive complaints (SCC), such as memory problems, concentration difficulties and slowed thinking, are some of the most common persistent (≥ 3 months) self-reported mTBI sequalae (Machamer et al., 2022; Theadom et al., 2016). SCC in mTBI result in substantial personal and societal burden through reduced the quality of life (Voormolen et al., 2019b), and increased healthcare use (Maas et al., 2017; Te Ao et al., 2014).

Persistent SCC in mTBI remain, despite their impact and clinical relevance, poorly understood. SCC occur frequently in general population (Iverson & Lange, 2003; Voormolen et al., 2019a) as well as in individuals with non-head injury trauma (Meares et al., 2011) or chronic pain (Roth et al., 2005). Surprisingly little is known about the relative specificity of SCC in mTBI after the early recovery period. Some reports (Clarke et al., 2012; Wäljäs et al., 2015) suggest SCC may remain elevated in patients with mTBI compared to healthy controls at least 3 months following injury, but evidence from studies including non-head injury trauma controls is inconsistent (Levin et al., 2021; Meares et al., 2011; Ponsford et al., 2011).

Cognitive test performance deficits are often seen in the early recovery period following mTBI, but these typically resolve within 3 months (Iverson et al., 2019). Although SCC remain common among patients with mTBI even thereafter, many studies report a lack of clear association between SCC and test-based cognitive performance beyond this time point (Karr et al., 2019; Stenberg et al., 2020; Stillman et al., 2020; Stulemeijer et al., 2007). Thus, underlying cognitive deficit is unlikely to be the sole explanation for persistent SCC in mTBI. Patient-related vulnerability factors, such as psychological distress and fatigue, are considered to have a role in post-mTBI SCC (Chamelian & Feinstein, 2006; Levy et al., 2023; Stillman et al., 2020; Stulemeijer et al., 2007), but protective psychological factors have received less attention.

Psychological resilience refers to an individual’s ability to mentally recover and bounce back from adverse life events (Smith et al., 2010), and it contributes to lower SCC in healthy older adults (Miller et al., 2023), as well as in individuals with small vessel disease (Arola et al., 2021). As for mTBI, higher resilience is associated with lower fatigue (Losoi et al., 2014; Losoi et al., 2015) and overall post-concussion symptom burden (Losoi et al., 2015; Oldenburg et al., 2018). Associations with SCC specifically have not been, to the best of our knowledge, previously examined.

Another potential protective psychological factor is perceived social support, a construct that refers to an individual’s belief in the availability of support from others in their social network during the times of need (Barrera, 1986). Data from other populations (Kever et al., 2021; Weng et al., 2020) suggest an association between perceived social support and SCC levels, but this topic remains unexplored in the mTBI context.

The aim of the present study was to examine SCC in a prospectively recruited sample of working-age adult patients 3 months after mTBI. We compared SCC in patients with mTBI to orthopedic injury (OI) controls, and explored the associations between SCC, cognitive test performance and protective psychological factors in patients with mTBI.

## METHODS

### Setting

The present cross-sectional study is part of a larger prospective longitudinal research project on mTBI outcome carried out at the Traumatic Brain Injury Outpatient Clinic of Helsinki University Hospital, Helsinki, Finland between March 2015 and September 2018. The ethics committee of Helsinki University Hospital approved the study protocol, and all participants gave written informed consent for participation. All study procedures were conducted in accordance with the principles of the Declaration of Helsinki.

### Participants

**Fig. 1:**
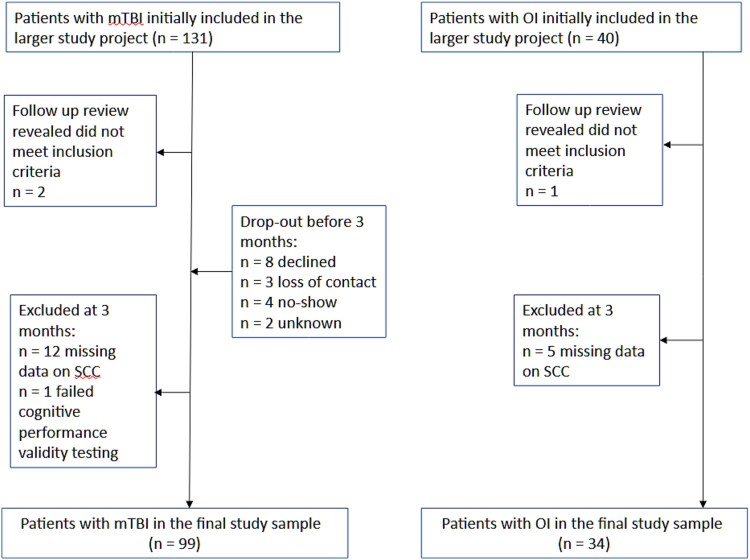
Diagram of the participant flow into the final study sample. MTBI, mild traumatic brain injury; OI, orthopedic injury; SCC subjective cognitive complaints.

Participants with mTBI were recruited from referrals to the Traumatic Brain Injury Outpatient Clinic of Helsinki University Hospital. Referrals were made for screening of further outpatient needs as part of clinical TBI management practice, not because of any specific symptoms or compensation issues. Participants were enrolled in the study within 12 days after sustaining injury*.* The definition of mTBI was based on the World Health Organization Collaborating Centre Task Force on Mild Traumatic Brain Injury criteria (Carroll et al., 2004), which include one or more following: (i) confusion or disorientation, loss of consciousness (LOC) for 30 min or less, post-traumatic amnesia (PTA) less than 24 hr, and/or other transient neurological abnormalities such as focal signs, seizure, and intracranial lesion not requiring surgery; and (ii) Glasgow Coma Scale score of 13–15 after 30 min or later upon presentation for healthcare. To control for nonspecific effects of traumatic injury and risk-taking behaviors that can predispose individuals to injury, an OI comparison group was also included. The OI group comprised patients with lower extremity injury recruited from the Trauma Emergency Department of Helsinki University Hospital. Due to patient availability and research logistics only patients with ankle fracture were included in the OI group. In the larger study project, exclusion criteria for both participant groups were as follows: Age under 18 years or over 68 years, previous diagnosis of schizophrenia, schizoaffective disorder, developmental disability, current alcohol or drug dependence, visual or hearing impairment, not being fluent in Finnish, or contraindication for magnetic resonance imaging (MRI). In addition, any suspicion of having sustained a head injury based on hospital records, patient interview, or MRI, was an additional exclusion criterion for the OI group. The present study also excluded all participants who did not complete the self-report measure on SCC or failed cognitive performance validity testing. The final sample comprised of 99 patients with mTBI and 34 orthopedic trauma controls. Figure 1 shows the participant flow in the study.

### Procedure

Information on clinical mTBI injury-related variables, including cause of injury and presence and length of LOC, PTA, and acute brain CT-scan results, were collected from emergency department patient records at the time of the study enrollment. All participants underwent brain structural MRI scanning (3 T, Siemens Magnetom Verio) as part of the study protocol, patients with mTBI 3 to 36 (median 10) days after injury, and patients with OI as soon after recruitment as was convenient for them. At ~3 months after injury (median 90 days, range 42–178 days) all participants underwent neuropsychological assessment and completed self-report measures on SCC, depressive symptoms, fatigue, pain, resilience, and perceived social support.

### Measures

#### Cognitive test performance

The neuropsychological test battery comprised 16 established cognitive tests, which were grouped into six functional cognitive domains to reduce the risk of false-positive results inherent in running multiple tests. *Processing speed* was evaluated with the Trail Making Test part A (time to complete) (Reitan, 1958), the Stroop color naming subtask (50 items, time to complete) (Lezak et al., 2012; Stroop, 1935), and the Coding subtest of the Wechsler Adult Intelligence Scale – Fourth Edition (WAIS-IV) (Wechsler, 2008). *Attention and working memory* was evaluated with the Digit Span Forward and Backward subtasks of the WAIS-IV (Wechsler, 2008). *Executive function* was evaluated with the Stroop Test color interference subtask (time to complete) (Lezak et al., 2012; Stroop, 1935), the Trail Making Test part B (time to complete) (Lezak et al., 2012; Reitan, 1958), and a phonemic fluency task (Lezak et al., 2012). *Immediate* and *delayed memory* were evaluated with the Logical Memory I and II, Word List I and II, and Visual Reproduction I and II subtests of the Wechler Memory Scale – Third Edition (WMS-III) (Wechsler 1997). Only Story A of the WMS-III Logical Memory subtest was used due to time constraints. *Reasoning* was evaluated with the Similarities and Block Design subtests of the WAIS–IV (Wechsler, 2008).

#### Cognitive performance validity testing

Cognitive performance validity was evaluated with two measures: Reliable Digit Span derived from the WAIS-IV Digit span subtest (Schroeder et al., 2012) and the Rey 15-Item Memory Test (Lezak et al., 2012). Cut-off score of ≤7 was used for the former and ≤ 8 for the latter (Lezak et al., 2012; Schroeder et al., 2012). Participants were considered to show suboptimal effort if they failed both these measures.

#### Self-report measures

##### Subjective cognitive complaints

were assessed with the cognitive subscale of the Rivermead post-concussion questionnaire (RPQ-COG) (King et al., 1995; Potter et al., 2006). This subscale comprises of the three cognitive RPQ items, namely: “forgetfulness, poor memory”, “poor concentration”, and “taking longer to think”. Respondents rate the presence and severity of each complaint over the past 24 hr relative to their experience of the same complaint prior to injury on a 5-point scale from 0 to 4 as follows: not experienced the symptom (0), no more of a problem (1), a mild problem (2), a moderate problem (3), and a severe problem (4). A total score is calculated by adding all items with a score greater than 1 (theoretical range 0–12).

##### Psychological resilience

was assessed with the 14-item abbreviated version of The Resilience scale (RS-14) (Wagnild & Young, 1993; Wagnild, 2016). Individual items include statements such as “When I’m in a difficult situation, I can usually find my way out of it” and “I usually manage one way or another”. Respondents state the degree to which they agree or disagree with each item on a 7-point Likert-type scale from 1 (strongly disagree) to 7 (strongly agree). A total score is calculated by adding all items (theoretical range from 14 to 98).

##### Perceived social support

was assessed with the abbreviated four-item version of the Medical Outcome Study Social Support Survey (MOS-SSS-4) (Gjesfjeld et al., 2008; Sherbourne & Stewart, 1991). Respondents rate how often they feel certain types of social support would be available to them if such support was needed (e.g., “If You needed it, how often there is someone to turn to for suggestions about how to deal with a personal problem”). Items are rated on a 5-point Likert scale from “none of the time” (1) to “all of the time” (5). Scores on each item are summed to give a total score ranging from 4 to 20.

##### Depressive symptoms

were assessed with the Beck Depression Inventory Fast Screen (BDI-FS) (Beck et al., 1997; Beck et al., 2000), an abbreviated version of the Beck Depression Inventory – II Edition (Beck et al., 1996). BDI-FS is specifically designed for patients with somatic medical conditions. It comprises the seven non-somatic items from the original BDI-II (sadness, pessimism, past failure, loss of pleasure, self-dislike, self-criticalness, and suicidal thoughts or wishes), thus tapping exclusively on the emotional and negative thought content aspects of depressive symptoms. Each item consists of four alternative statements. Respondents are asked to endorse the one that best describes how they are currently feeling. Items are scored on a scale from 0 to 3 and summed for a total score ranging from 0–21. Higher scores reflect greater symptom severity.

##### Fatigue

was assessed with The Barrow Neurological Institute Fatigue Scale (BNI-FS) (Borgaro et al., 2004) which is a self-report questionnaire designed to assess fatigue after brain injury. It comprises of 10 items rated on an 8-point scale as follows: Rarely a problem (0–1), occasional problem, but not frequent (2–3), frequent problem (4–5), a problem most of the time (6–7). Respondents indicate the extent to which each of the items has been a problem for them since their injury. A total score is calculated by adding all items (theoretical range 0–70).

##### Pain

was assessed with Pain Visual Analog Scale (PVAS), a widely used brief measure of pain intensity (Huskisson, 1974). PVAS consists of a 10-cm-long straight line with endpoints defining extreme limits to pain intensity (from “no pain” to “maximum pain ever experienced”). Respondents indicate their current level of pain by placing a mark along the line. Score was determined by measuring the distance (mm) on the line between the “no pain” endpoint and the responder’s mark (theoretical range 0–100).

### Statistical analyses

The Statistical Package for Social Sciences (IBM SPSS Statistics for Windows, Version 29.0, Armonk, NY, United States: IBM Corporation) was used for all statistical analyses. Missing data were not imputed. Continuous data are presented as means with standard deviations (normally distributed variables) or medians and range (nonnormally distributed variables), and categorical data as numbers and percentages. Group comparisons for demographical, injury related, and self-report questionnaire variables were performed using Pearson’s chi-square test, Fisher’s exact test, or Fisher–Freeman–Halton exact test for binary categorical variables, Student’s t test for normally distributed continuous variables, and Mann–Whitney U test for nonnormally distributed continuous variables. Group differences in cognitive test performance were examined with multivariate analyses of variance (MANOVAs) and univariate analyses of variance (ANOVAs). First, each cognitive domain was analyzed with a separate MANOVA. If the MANOVA was significant, the individual cognitive performance measures included in the domain were further analyzed with subsequent ANOVAs. For cognitive test performance variables, logarithmic transformations to obtain normality were used when needed, but untransformed means are reported. The level of significance was set at *p* < .05, and Bonferroni-adjusted p values are reported for univariate follow-up comparisons (ANOVAs). For estimates of effect size, phi for Pearson’s chi-square test, Cohen’s d for t-test, r for the Mann–Whitney U, and partial eta squared (ηp^2^) for analysis of variance were used. Descriptions of effect sizes were based on Cohen (1988) as follows; Cohen’s d: small >0.2, medium >0.5, large >0.8), phi: small >0.1, medium >0.3, large >0.5, r: small >0.1, medium >0.3, large >0.5 and ηp^2^: small >0.01, medium >0.06, large 0.14.

## RESULTS

### Sample characteristics

For the patients with mTBI, ground level fall (30.3%) was the most common cause of injury, being followed by bicycle accidents (24.2%), falls from the heights (21.2%), sports (11.1%), motor vehicle or pedestrian traffic accidents (8.1%), and other (5.1%). Eighty-eight (88.9%) of the patients with mTBI had PTA, with the median duration being 87 min (range 1–1440 min). Fifty-seven (57.6%) patients with mTBI had witnessed LOC, 17 (17.2%) had no LOC, and for 25 (25.3%) information on LOC was missing or uncertain. Duration of LOC was available for 51 of the 57 patients with LOC, and it was 10 min or less in all cases (median 1 min, range 1–10 min). Forty-one (41.4%) of the patients with mTBI had trauma-related intracranial abnormality in CT (n = 27) or MRI (n = 41). The mTBI group included three patients (3%) who were involved in litigation.

There were no statistically significant differences between the mTBI and OI groups in age (mean [M] = 40.2, standard deviation [SD] = 13.2 vs. M = 42.4, SD = 11.8, t(131) = −0.840 *p* = .402), education (M = 15.9, SD = 3.6 vs. M = 15.9, SD = 3.4, t(131) = −0.089, *p* = .929), or gender (50.5% vs. 50.0%, female, Χ^2^(1) = 0.959, *p* = 1.000). Ninety (90.9%) of the patients with mTBI and 33 (97.1%) of the OI controls had been working or studying prior to their injury, and 85 (94.4%) and 28 (84.8%) had returned to these activities by the time of the 3-month assessment, respectively.

The mTBI and OI groups did not differ significantly in cognitive test performance, and this remained unchanged when age, gender, and education were included as covariates. The two groups did not differ in depressive symptoms, fatigue or pain (Table 1). Likewise, there were no significant differences between the two groups in psychological resilience (median [Md] = 85, range 19–98 vs. Md = 81, range 56–97, U = 1352.5, *p* = .088) or perceived social support (Md = 20, range 6–20 vs. Md = 20, range 11–20, U = 1651.0, *p* = .932).

**Table 1:** Cognitive performance, depressive symptoms, fatigue, and pain for patients with mild traumatic brain injury and orthopedic injury controls

	**mTBI (n = 99)**	**OI (n = 34)**		
**Cognitive performance**	M (SD)		*p* value	ηp2
*Processing speed*			.936	0.003
WAIS- IV Coding	74.4 (14.9)	73.5 (12.0)		
Stroop color naming, s^a^ ^b^	36.6 (8.8)	37.5 (8.3)		
TMT A, s^a^	28.4 (9.4)	28.8 (10.4)		
*Executive function*			.862	0.006
TMT B, s^a^ ^b^	65.6 (22.9)	68.0 (21.9)		
Phonemic fluency	33.3 (8.5)	32.4 (9.0)		
Stroop color inference, s^a^ ^b^	55.8 (13.4)	55.9 (11.1)		
*Attention and working memory*			.995	0.000
WAIS-IV Digit Span forward (total score)	9.3 (2.1)	9.4 (2.1)	.927	0.000
WAIS-IV Digit Span backward (total score)	9.4 (2.4)	9.5 (2.5)	.940	0.000
*Immediate memory*			.236	0.032
WMS-III Logical Memory I (1. story)^b^	16.2 (2.7)	16.5 (3.6)		
WMS-III Word Lists I^b^	33.3 (5.8)	35.1 (5.8)		
WMS-III Visual Reproduction I	89.7 (11.5)	88.6 (10.3)		
*Delayed memory*			.466	0.020
WMS-III Logical Memory II (1. story)^b^	15.9 (3.7)	16.4 (3.5)		
WMS-III Word Lists II^b^	8.2 (2.5)	8.5 (2.5)		
WMS-III Visual Reproduction II	62.2 (21.9)	58.8 (26.4)		
*Reasoning*				
WAIS-IV Block Design	51.8 (9.4)	47.9 (11.0)	.283	0.019
WAIS-IV Similarities	27.8 (3.1)	27.1 (3.4)		
**Self-report measure**	Md (range)		*p* value	r
Depressive symptoms (BDI-FS)	0 (0–11)	0 (0–6)	.544	−0.053
Fatigue (BNI-FS)	5 (0–50)	9.5 (0–51)	.123	−0.134
Pain (PVAS)	3 (0–76)	6 (0–58)	.246	−0.101

*Note.* For the cognitive performance, p-values are from MANOVAs and subsequent ANOVAs, and for the self-report measures from Mann–Whitney U-tests.

^a^A logarithmic transformation was used in the analyses. Untransformed means are reported for all variables.

^b^Data missing for 1 participant in the mTBI group. BDI-FS, Beck Depression Scale Fast Screen; BNI-FS, Barrow Neurological Institute Fatigue Scale; mTBI, mild traumatic brain injury; OI, orthopedic injury; PVAS, Pain Visual Analog Scale TMT, Trail Making Test; WAIS-IV, Wechler Adult Intelligence Scale – Fourth Edition; WMS-III, Wechler Memory Scale- Third Edition.

### Subjective cognitive complaints in mTBI and OI

Median RPQ-COG score was 0 (range 0–10) for the mTBI group and 0 (range 0–6) for the OI group. In both groups, most endorsed individual complaint was poor concentration (mTBI n = 18 [18.2%] vs. OI n = 5 [14.7%]). Seventy-two (72.7%) of the patients with mTBI reported no remaining cognitive complaints, twenty-one (21.2%) reported at least one mild complaint, and further six (6.0%) at least one moderate to severe complaint (moderate n = 5, severe n = 1). In the OI group, at least one mild complaint was endorsed by six (17.6%) of the patients, and none endorsed moderate or severe complaints. The mTBI and OI groups did not differ significantly in their SCC endorsement (Table 2).

**Table 2:** Subjective cognitive complaints for patients with mild traumatic brain injury and orthopedic injury controls

	**mTBI (n = 99)**	**OI (n = 34)**	**p-value**
RPQ-COG total score, Md (range)†	0 (0–10)	0 (0–6)	0.232
RPQ-COG individual item endorsement n (%)			
Forgetfulness, poor memory‡	17 (17.2)	3 (8.8)	0.282
Poor concentration‡	18 (18.2)	5 (14.7)	0.795
Taking longer to think‡	15 (15.2)	2 (5.9)	0.236
Severity of cognitive complaints, n (%)§			0.568
None	72 (72.7)	28 (82.4)	
Mild	21 (21.2)	6 (17.6)	
Moderate	5 (5.1)	0 (0)	
Severe	1 (1.0)	0 (0)	
RPQ-COG >2, n (%)¶	27 (27.3)	6 (17.6)	0.358

*Note.* P-values are from Mann–Whitney U-test (†), Fisher’s Exact tests (‡), Fisher–Freeman–Halton exact test (§), or Pearson Pearson’s chi-square test (¶). mTBI: mild traumatic brain injury; OI: orthopedic injury; RPQ-COG: Rivermead post-concussion questionnaire cognitive subscale.

### Comparison of patients with mTBI with and without SCC

Within the mTBI group, there were no statistically significant differences between the SCC and non-SCC subgroups in any demographic or injury-related characteristics (Table 3). Of the five participants with mTBI who were still on sick leave at the time of the assessment, two belonged to the SCC group and three to the non-SCC group.

**Table 3:** Demographic variables, injury characteristics and cognitive performance for patients with mild traumatic brain injury with and without subjective cognitive complaints

**Variable**	**SCC (n = 27)**	**Non-SCC (n = 72)**	***p* value**	**Effect size**
**Demographic characteristics**				
Age, years†	39.6 (13.7)	40.5 (13.0)	.765	0.068
Gender, female, n (%)‡	14 (51.9)	36 (50.0)	1.000	0.016
Education, years†	16.1 (3.1)	15.8 (3.7)	.670	−0.096
**Injury characteristics**				
Witnessed LOC, n (%)‡^a^	17 (77.3)	40 (76.9)	1.000	0.004
Length of LOC, Md (range), min§^b^	1 (1–10)	1 (1–10)	.776	−0.040
PTA, yes, n (%)‡	24 (88.9)	64 (88.9)	1.000	0.000
Length of PTA, Md (range), min §	120 (1–1440)	65 (1–1440)	.944	−0.008
Traumatic lesions in CT, n (%)‡^c^	8 (32.0)	19 (27.1)	.797	0.047
Traumatic lesions in MRI, n (%)‡	15 (55.6)	26 (36.1)	.109	0.176^*^
**Cognitive performance**				
*Processing speed* ¶			.853	0.008
WAIS- IV Coding	73.3 (13.3)	74.8 (15.5)		
Stroop color naming, s#d	37.7 (9.1)	36.2 (8.7)		
TMT A, s#	29.9 (11.5)	27.8 (8.5)		
*Executive function* ¶			.306	0.038^*^
TMT B, s#	74.1 (30.4)	62.3 (18.7)		
Phonemic fluency	31.5 (7.8)	33.9 (8.7)		
Stroop interference, s#	59.0 (14.5)	54.7 (12.9)		
*Attention and working memory*¶			**.009**	0.093^**^
Digit Span forward total score	8.4 (2.2)	9.7 (2.0)	**.014**	0.073^**^
Digit Span backward total score	8.3 (2.1)	9.9 (2.4)	**.01**	0.079^*^
*Immediate memory*¶			.324	0.036^*^
WMS-III Logical Memory I (1. story)^d^	16.0 (3.0)	16.3 (2.6)		
WMS-III Word Lists I^d^	31.6 (5.4)	33.9 (5.9)		
WMS-III Visual Reproduction I	86.9 (14.1)	90.8 (10.3)		
*Delayed memory*¶			.551	0.022^*^
WMS-III Logical Memory II (1. story)^d^	15.4 (4.0)	16.1 (3.5)		
WMS-III Word Lists II^d^	7.9 (2.3)	8.3 (2.5)		
WMS-III Visual Reproduction II	57.3 (26.1)	64.1 (19.9)		
*Reasoning*¶			.360	0.021^*^
WAIS-IV Block Design	49.4 (10.5)	52.7 (8.9)		
WAIS-IV Similarities	27.4 (3.6)	28.0 (2.9)		

*Note.* Data are presented as mean (SD) unless otherwise stated. P-values are from independent samples t-tests (†), Pearson khii-square test (‡), Mann–Whitney U-test (§), or MANOVAs followed by ANOVAs (¶). Bonferroni adjusted p-values are reported for univariate follow-up analyses (ANOVAs).

^#^A logarithmic transformation was used in the analyses. Untransformed means are reported for all variables. Significant p-values are bolded. Effect sizes are Cohen’s d (^*^ small >0.2, ^**^ medium >0.5), phi (^*^small >0.1, ^**^ medium >0.3,), r (^*^ small >0.1, ^**^ medium >0.3) or ηp2 -values (^*^small >0.01, ^**^medium >0.06) according to Cohen (1988). LOC, loss of consciousness; MRI, magnetic resonance imaging; mTBI, mild traumatic brain injury; OI, orthopedic injury; PTA, posttraumatic amnesia; SCC, subjective cognitive complaints; TMT, Trail Making Test; WAIS-IV, Wechler Adult Intelligence Scale – Fourth Edition; WMS-III, Wechler Memory Scale- Third Edition.

^a^Data missing for five participants in SCC and 20 in non-SCC group.

^b^Data missing for three participants in SCC and three in non-SCC group.

^c^Data missing two participants the SCC and two in non-SCC group.

^d^Data missing for 1 participant in the non-SCC group.

Table 3 shows a comparison of the cognitive test performance between the SCC and non-SCC groups. There was a significant difference in the attention and working memory domain between the two groups (*p* = .009). Subsequent univariate analyses indicated that the SCC group had significantly lower scores on both Digit Span Forward and Backward subtasks of WAIS-IV compared to the non-SCC group (*p* = .014 and *p* = .01, respectively). The SCC and non-SCC groups did not differ significantly in the domains of processing speed, executive function, immediate memory, delayed memory, or reasoning (Table 3). Inclusion of age, gender, and education as covariates did not change any of these results.

As shown in Table 4, patients in the SCC group reported significantly lower psychological resilience (*p* = .005) and perceived social support (*p* = .009) compared to the non-SCC group. The SCC and non-SCC groups also differed significantly in their endorsement of depressive symptoms (*p* < .001) and fatigue (*p* < .001). The two groups did not differ in self-reported pain.

**Table 4:** Self-report measures for patients with mild traumatic brain injury with and without subjective cognitive complaints

Variable	SCC (n = 27)	Non-SCC (n = 72)	*p* value	Effect size
Psychological resilience (RS-14)	77 (48–98)	86.5 (19–98)	**.005**	−0.282^*^
Perceived social support (MOS-SSS-4)^a^	18 (10–20)	20 (6–20)	**.009**	−0.265^*^
Depressive symptoms (BDI-FS)	1 (0–11)	0 (0–5)	**<.001**	−0.443^**^
Fatigue (BNI-FS)	18 (6–50)	3 (0–46)	**<.001**	−0.611^***^
Pain (PVAS)	5 (0–66)	2 (0–76)	.095	−0.168^*^

*Note.* Data are presented as median (range). P-values are from Mann–Whitney U-tests. Significant p-values are bolded. Effect sizes are r-values (^*^ small >0.1, ^**^ medium >0.3, ^***^large >0.5). BDI-FS, Beck Depression Scale Fast Screen; BNI-FS, Barrow Neurological Institute Fatigue Scale; MOS-SSS-4, abbreviated Medical Outcome Study Social Support Survey; PVAS, Pain Visual Analog Scale; RS-14, The Resilience Scale, 14-item version; SCC, subjective cognitive complaints.

^a^Data for one participant missing in non-SCC group.

## DISCUSSION

The present study examined SCC 3 months after injury in a prospective recruited sample of working-age adult patients with mTBI, and explored the associations between SCC, cognitive test performance and psychological factors.

The proportion of patients with mTBI endorsing at least some SCC in RPQ-COG 3 months post-injury was 27.3%, which is very close to the 26.5% previously reported by Stenberg et al. (2020). It is noteworthy that 17.6% of the OI controls also endorsed SCC, and the mTBI and OI groups did not differ significantly in terms of SCC in our study. These findings support the notion that mild SCC is relatively common and unspecific. However, it is noteworthy that while SCC reported by the OI controls was at most mild, a proportion of the patients with mTBI reported moderate-to-severe SCC. The extremely small number of these patients (n = 6) prevents further exploration of this issue in our sample, but nevertheless, this observation highlights the heterogeneity of self-reported mTBI recovery.

Within the mTBI group, the neuropsychological test performance of those with SCC was comparable to that of those without SCC in majority of the cognitive domains examined. The one exception to this was the attention and working memory domain, in which SCC group had significantly lower scores than the non-SCC group in both the Digit Span Forward and Backward subtasks of WAIS-IV. This finding needs to be interpreted with caution in our study sample, but some previous studies (Carter-Allison et al., 2016; Suhr & Gunstad, 2005) suggest factors such as negative expectations about the potential effects of head injury (“diagnosis threat”) can affect attention and working memory performance in mTBI.

Overall, our findings align with those previous studies that have found mainly nonsignificant associations between SCC and cognitive test performance in mTBI (Karr et al., 2019; Stenberg et al., 2020; Stillman et al., 2020; Stulemeijer et al., 2007). There are several possible explanations for this lack of association. Many non-injury factors as such as psychological distress (Chamelian & Feinstein, 2006; Levy et al., 2023; Stillman et al., 2020; Stulemeijer et al., 2007), negative recovery expectations (Mah et al., 2018), and the good-old-days bias (Gunstad & Suhr, 2001) are believed to have a role in symptom reporting after mTBI. However, it has also been suggested that patients with mTBI may be able compensate for subtle cognitive deficits by increased effort, thus maintaining unimpaired cognitive test performance but at the expense of excessive energy expenditure leading to fatigue (Cantor et al., 2013). From this perspective it is interesting that, in our study sample, patients with mTBI with SCC endorsed more self-reported fatigue compared to those without SCC. The further interpretation of this finding is, however, beyond the scope of this study. Another potential explanation is that this study employed traditional neuropsychological cognitive performance measures, whose sensitivity to detect subtle cognitive deficits in mTBI has sometimes been debated (Bigler et al., 2013). It is unlikely, however, that our results are wholly reducible to measurement issues, as some studies employing proposedly more sensitive computer-based methods with increasing task load, have similarly reported disassociation between SCC and cognitive test performance (Levy et al., 2024).

Our findings suggest that protective psychological factors may have a role in post-mTBI SCC. Patients with mTBI with SCC reported lower resilience and perceived social support compared to those without SCC. Whilst resilience is known to contribute to overall post-concussion symptom experience in mTBI (Losoi et al., 2015; McCayley et al., 2013; Oldenburg et al., 2018), our report adds to the existing evidence by suggesting it may also relate to SCC specifically. Furthermore, our findings on the association between perceived social support and SCC are interesting, as this issue has not been, to the best of our knowledge, previously explored in the mTBI context. As both psychological resilience and perceived social support reflect cognitive appraisal processes, they are, at least potentially, modifiable. Thus, they could provide targets for interventions for patients with SCC (but no cognitive test performance deficits) following mTBI. Further research, however, is still needed.

The strengths of our study include the clinically and radiologically well characterized mTBI group, and comprehensive neuropsychological assessment. Some important limitations need also to be considered. Our cross-sectional study design precluded us from drawing any conclusions about directionality or causality. The sample size was relatively small. This prevented us from exploring the group differences between patients with and without SCC in the OI group. Thus, further studies are needed to clarify whether the associations between SCC and lower perceived social support and psychological resilience found in this study are mTBI specific or also found in other injury groups. Patients with mTBI in our study were recruited from referrals to an mTBI outpatient clinic, and the proportion of MRI-positive patients was high. Thus, our study sample is skewed towards the more severe end of injury severity spectrum in mTBI. Also, patients in our study sample were relatively highly educated, and the proportion of those who had returned to work was high. Thus, our findings may not be generalizable to other mTBI populations. This study included a small number of patients with mTBI (n = 3) who were involved litigation. However, removing these individuals from the analyses did not significantly change any of our results. The sensitivity of the performance validity measures included in this study may not be optimal. Although this is one limitation of our study, we believe it is unlikely that this has caused any major bias. As already mentioned, the number of litigating patients was very small. More importantly, the mTBI and OI groups did not differ significantly in their cognitive test performance in our study, making it less likely that undetected suboptimal effort has substantially affected our results.

## CONCLUSIONS

This study provides support for the notion that persistent SCC following mTBI is not consistently related to cognitive test performance deficits. Furthermore, our findings add to the understanding of the role of psychological factors in mTBI recovery by suggesting SCC may associate with perceived social support and psychological resilience. These findings highlight the importance of considering psychological factors in the clinical assessment and intervention planning of patients presenting with SCC after mTBI.
